# Retraction Note: Circ_002117 binds to microRNA-370 and promotes endoplasmic reticulum stress-induced apoptosis in gastric cancer

**DOI:** 10.1186/s12935-022-02826-1

**Published:** 2022-12-13

**Authors:** Nan Zhou, Hui Qiao, Miaomiao Zeng, Lei Yang, Yongning Zhou, Quanlin Guan

**Affiliations:** 1 Department of the First Clinical Medical College, Lanzhou University, Lanzhou, 730000 People’s Republic of China; 2 Department of Medical Oncology, The First Hospital of Lanzhou University, Lanzhou, 730000 People’s Republic of China; 3 Department of Surgery, The First Hospital of Lanzhou University, Lanzhou, 730000 People’s Republic of China; 4 Department of Gastroenterology, The First Hospital of Lanzhou University, Lanzhou, 730000 People’s Republic of China; 5 Key Laboratory for Gastrointestinal Disease of Gansu Province, The First Hospital of Lanzhou University, Lanzhou, 730000 People’s Republic of China; 6 Department of Oncology Surgery, The First Hospital of Lanzhou University, No. 1, Donggang West Road, Chengguan District, Lanzhou, 730000 Gansu People’s Republic of China

**Retraction: Cancer Cell Int (2020) 20:465** 10.1186/s12935-020-01493-4

The Editors in Chief have retracted this article. After publication it was noted that there were irregularities in Figs. 3I and 5H that have led to a loss of confidence in the integrity of the results of this article. Guan Quanlin does not agree to this retraction. None of the other authors have responded to any correspondence from the Publisher about this retraction.

